# DNA Repair and Immune Response: Editorial

**DOI:** 10.3390/biom13010084

**Published:** 2022-12-30

**Authors:** Valentyn Oksenych

**Affiliations:** Institute of Clinical Medicine, University of Oslo, 0318 Oslo, Norway; valentyn.oksenych@medisin.uio.no

Developing B and T lymphocytes requires programmed DNA double-strand breaks followed by the activation of the DNA damage response (DDR) pathway and DNA repair. It is necessary for the DNA recombination process during the V(D)J recombination in maturating B and T lymphocytes to ensure the final assembly of the B cell receptor and T cell receptor genes [1,2,3,4,5,6,7]. The primary DNA repair process controlling V(D)J recombination is non-homologous DNA end-joining (NHEJ). Two additional processes specific to B cell maturation include DNA lesions and DNA repair. One is class-switch recombination (CSR), which is required to change the immunoglobulin class that also involves NHEJ and base excision repair (BER). The somatic hypermutation (SHM) process helps to increase the specificity of immunoglobulins (antibodies) and depends on different DNA repair pathways (BER and mismatch repair, MMR) [1,2].

There are several known NHEJ factors, but the extensive genetic interaction between the NHEJ-NHEJ, NHEJ-DDR, and DDR-DDR genes suggests that additional NHEJ and DDR proteins will be discovered, and currently, their functions are not known due to the complex compensatory mechanisms of the other factors. One central NHEJ gene with multiple known genetic interactions encodes an X-ray repair cross-complementing 4-like factor (XLF). It interacts genetically with several NHEJ factors, i.e., DNA-dependent protein kinase, catalytic subunit (DNA-PKcs) [8,9,10], a paralogue of XRCC4 and XLF (PAXX) [2,10,11,12,13,14,15,16,17,18,19,20], and a Modulator of retrovirus infection (MRI) [12,21,22]. Moreover, XLF has overlapping or complementary functions (functional redundancy or interacts genetically) with several DDR factors, i.e., Ataxia telangiectasia mutated (ATM) [23], histone H2AX [23], a mediator of DNA damage checkpoint 1 (MDC1) [24], p53-binding protein 1 (53BP1) [25,26], Really interesting new gene (RING) finger protein 8 (RNF8) and RNF168 [27], and Shieldin [28]. 

In the current Topical Collection, Raquel Gago-Fuentes et al. published a research article focusing on the genetic interaction between the NHEJ factors XLF, PAXX, and DNA-PKcs during the development of the central nervous system in mice. In particular, neural stem and progenitor cell populations were isolated from mice lacking one or two NHEJ factors. Using self-renewal capacity and differentiation and proliferation assays, it was concluded that XLF, PAXX, and DNA-PKcs are required for the early stages of neurodevelopment in mammals [29].

Richard Frock et al. published a Review article on the DNA repair process [30]. In addition to classical NHEJ, this review also focuses on alternative end-joining (A-EJ, see also [31]), and the DNA repair pathway choice in the G0/G1 phases of the cell cycle, especially in developing a B lymphocyte research model in the context of the recombination-activating gene (RAG)-mediated DNA breaks [30].

Valentyn Oksenych et al. published a research article on the roles of acetyltransferases General control nondepressible 5 (GCN5, or lysine acetyltransferase 2A, KAT2A) and p300/CBP-associated factor (PCAF, or KAT2B) in B cell development in mice [32]. Using a genetically modified mouse model combining germline knockout, conditional knockout, and knock-in mutations, as well as an overall loss-of-function strategy, the authors found that both GCN5 and PCAF are necessary for the B lymphocyte maturation in vivo. The count of mature B cells in mice lacking GCN5 was significantly reduced compared to the wild-type (WT) controls. The combined inactivation of GCN5 and PCAF resulted in even more dramatic effects observed in the spleen, blood, and bone marrow with even lower numbers of B cells when compared to single knockouts. Moreover, GCN5 is required for the immunoglobulin (Ig) class switch recombination process ex vivo, and the switching from IgM to IgG3 was significantly reduced in B cells lacking GCN5 protein [32]. Further studies are required to elucidate the mechanisms underlying the functions of GCN5 and PCAF in B cell development.

Sucheng Zhu et al. published a research article on the development of new methods to detect DNA modification [33]. The article focuses on three methods based on iodine-induced specific DNA cleavage. The new methods are dedicated to saving time and labor for the detection of DNA phosphorothioate modifications [33].

Finally, Pavlo Petakh et al. published a review article on Weil’s disease [34]. Leptospirosis is a zoonotic disease causing 60,000 deaths annually. The review describes immunopathogenesis, the influence of cytokines and genetic susceptibility on the course of the disease, and the role of gut microbiota in the clinical course. Additionally, it suggests that modulating the gut microbiota by probiotics or fecal microbiota transplantation is a possible area of further scientific research [34].

Thus, the Topical Collection highlighted the recent discoveries in DNA repair, lymphocyte maturation, development of novel methods, and immune response during disease. Additional DNA repair factors with roles in the development of the immune and nervous systems, as well as protection from genomic instability and cancer, will be discovered in the future, for example, by taking into consideration a complex genetic interaction between the DNA repair genes and the pathways in mammalian cells.
